# Transcriptome analyses reveal key roles of alternative splicing regulation in atlantic salmon during the infectious process of Piscirickettsiosis disease

**DOI:** 10.1016/j.heliyon.2023.e22377

**Published:** 2023-11-17

**Authors:** Scarleth Bravo, Javier Moya, Francisco Leiva, Osiel Guzman, Rodrigo Vidal

**Affiliations:** a Laboratory of Genomics, Molecular Ecology and Evolutionary Studies, Department of Biology, Universidad de Santiago de Chile, Santiago, Chile; b Benchmark Animal Health Chile, Santa Rosa 560 of.26, Puerto Varas, Chile; c IDEVAC SpA, Francisco Bilbao 1129 of. 306, Osorno, Chile

**Keywords:** Piscirickettsiosis, RNA-Seq, Atlantic salmon, Alternative splicing

## Abstract

In the Chilean salmon farming industry, infection by *Piscirickettsia salmonis* is the primary cause of the main bacterial disease known as Piscirickettsiosis, which has an overwhelming economic impact. Although it has been demonstrated that Piscirickettsiosis modifies the expression of numerous salmonids genes, it is yet unknown how alternative splicing (AS) contributes to salmonids bacterial infection. AS, has the potential to create heterogeneity at the protein and RNA levels and has been associated as a relevant molecular mechanism in the immune response of eukaryotes to several diseases. In this study, we used RNA data to survey *P. salmonis*-induced modifications in the AS of Atlantic salmon and found that *P. salmonis* infection promoted a substantial number (158,668) of AS events. Differentially spliced genes (DSG) sensitive to Piscirickettsiosis were predominantly enriched in genes involved in RNA processing, splicing and spliceosome processes (e.g., hnRNPm, hnRPc, SRSF7, SRSF45), whereas among the DSG of resistant and susceptible to Piscirickettsiosis, several metabolic and immune processes were found, most notably associated to the regulation of GTPase, lysosome and telomere organization-maintenance. Furthermore, we found that DSG were mostly not differentially expressed (5–7 %) and were implicated in distinct biological pathways. Therefore, our results underpin AS achieving a significant regulatory performance in the response of salmonids to Piscirickettsiosis.

Aquaculture has a great deal of potential to expand to meet human needs for seafood products. Atlantic salmon (*Salmo salar*) is one the main economic contributors to the marine finfish aquaculture sector [1]. However, infectious diseases have involved Atlantic salmon aquaculture around the world, affecting its sustainability and growth [2]. One of the main infectious diseases in salmonid aquaculture is Piscirickettsiosis, with almost 50 % of the mortality caused by the disease in Chilean Atlantic salmon farmed in 2021, attributable to this disease (http://www.sernapesca.cl/). Nevertheless, resistant Atlantic salmon families have been reported [3]. Even though several non-antibiotic strategies have been developed against Piscirickettsiosis (e.g., vaccines, genetic selection, natural products), they clearly have not been as effective as expected in the field [4]. The causal agent of Piscirickettsiosis is *Piscirickettsia salmonis*, a facultative gram-negative intracellular bacterium [5]. The most relevant initial clinical signs of Piscirickettsiosis are chronic sepsis, anemia and skin lesions and in its late stage, a distinguishable necrotization of fundamental organs, such as the kidney [6]. The kidney, is an admitted organ with pivotal immune and excretory functions in teleosts. In addition, the anterior (head) kidney is a region with a widely recognized haemotopoietic role, comparable to bone marrows in higher metazoans. Although it is accepted that macrophages are apparently the mayor target cell, where *P. salmonis* replicates and survives, the molecular process of infection is not totally well known [7], which will elucidate the relative effectiveness of the strategies developed to combat Piscirickettsiosis. Previous transcriptomic studies have been conducted on Atlantic salmon to understand their response to *P. salmonis* [[8], [9], [10], [11], [12], [13], [14]]. These investigations have exposed to light important elements of the early response to infection, such as heightened inflammation, modification of type 1 T helper cell T (e.g., interferon-gamma) activity, and restriction in the processing and display of antigens. Although these studies shed light on the underlying molecular basis of the Atlantic salmon transcriptome response to *P. salmonis*, the functioning or importance of mechanisms that regulate gene expression through RNA processing, like alternative splicing, remains unclear. Alternative splicing (AS) is a critical post-transcriptional process in metazoans that generates different messenger RNAs (mRNAs) (isoforms) from a single gene by selectively removing or retaining exons/introns from immature mRNAs. mRNA and protein isoforms produced by AS of pre-mRNAs may differ in its subcellular localization, stability, structure and function, resulting in gene expression regulation [15]. Hence, AS contributes to eukaryotes gene transcriptional heterogeneity and activity. Over 90 % of multiple exon genes undergo AS in humans [16] and the frequency is a high as 82.76 % in teleosts [17]. Although several components participate in the AS process regulation, this is normally directed by both cis specific elements and trans acting RNA binding proteins (e.g., heterogeneous nuclear ribonucleoproteins and splicing factors). According to the splicing pattern, AS can be associated to five aim categories, alternative 3′ splice sites (A3SS), skipped exon (SE), alternative 5′ splice sites (A5SS), mutually exclusive exons (MXE) and intron retention (IR) [18]. Numerous studies have described a fundamental role of AS processes in the response of terrestrial animals to bacterial, viral, and endoparasite infections [[19], [20], [21], [22]]. Moreover, the role of AS in disease resistance has been described for both plants and animals [23,24]. In contrast, comprehensive studies of pathogen-responsive AS, such as bacteria, are scarce in fish. Two recent studies, using a genome-wide approach, have demonstrated the role of differential splicing in the immune host response following gram-negative bacterial infection in the gill tissue of channel catfish (*Ictalurus punctatus*) [25,26]. Both studies found an increase in the frequency of AS after infection, as well as an enrichment of genes associated with immune, RNA splicing and RNA binding pathways. Since no genome-wide studies of the splicing process in salmonids in response to bacterial pathogens, have been performed it is unknown whether differential AS is a relevant response of salmonids to Piscirickettsiosis. Therefore, in the current study we used RNA-seq datasets, to perform a comparative genome-wide analysis of differential AS from control and infected *P. salmonis* Atlantic salmon head kidney tissue at two different time points, and then identified DASE (differential alternative splicing events) associated with susceptible and resistant phenotypes, to determine the role and contribution of AS in the Atlantic salmon response to Piscirickettsiosis. Our results revealed a significant presence of differential AS in Atlantic salmon after *P. salmonis* infection, and advance our understanding of the biological relevance of AS in salmonids immune responses.

## 1 Materials and methods

### 1.1 RNA-seq datasets, alignment and transcript assembly

To investigate differential AS of *P. salmonis*-infested Atlantic salmon from a global and specific resistant/susceptible phenotypes context, we downloaded the RNA-seq datasets Sequence Read Archive No. PRJNA669807 [27] of Atlantic salmon from the National Center for Biotechnology Information (NCBI). Briefly, this dataset comprises 59 head kidney tissues transcriptomes, ordered into control (pre-challenged) and *P. salmonis* (strain LF-89) challenged pre-smolts fish (average weight 135 ± 47 g) at two different time points, 3 and 9 days post-infection (dpi) (Table S1). Prior to the challenge, fish were screened to principal pathogens (e.g., ISA, IPNV, Mycoplasma, etc). Besides, a subset of the Atlantic salmon organisms of this dataset has a related phenotypic metric (EBV; Estimated Breeding Value) of Piscirickettsiosis resistance or susceptibility (Table S1). Raw reads were trimmed for low quality and adapter with Fastp v 0.11.8 [28]. The R package STAR v 2.7.10a (two-pass model activated) [29] was used to align the trimmed sequence reads to the Atlantic salmon reference genome (Ssal v3.1; NCBI Accession No. GCF 905237065.1). ComBat-seq [30], was utilized for batch effect adjustment. The TranscriptomeSAM option and the R package RSEM v 1.3.3 [31] were utilized to assess the levels of transcript/isoform expression.

### 1.2 Detection of head kidney alternative splicing events (ASE) and DASE

rMATS v 4.1.1 [32], with default parameters, was used for ASE identification, classification, and DASE analysis. For ASE to be identified, a minimum of 10 reads uniquely mapped in splice junctions were required. The retrieved ASE were divided into the main AS categories: A5SS, A3SS, SE, IR, and MXE. DASE were calculated using the recognized differential Percent-spliced-in (PSI) and those with |ΔPSI| ≥10 % and with a p-value false discovery rate (FDR) (Benjamini and Hochberg correction) < 0.01 were considered as statistically significant. DASE genes were identified as differentially spliced genes (DSG). Low expression isoforms/transcripts were removed if at least five samples had a Relative Log Estimate less than 0.5. To assort PSI patterns in consideration of infection times and phenotypes, a heatmap cluster analysis was performed using complete clustering on euclidian distances, with Pheatmap v-1.0.12 (https://cran.r-project.org/web/packages/pheatmap/).

### 1.3 Differential gene expression

To evaluate the relationship among AS and transcriptional patterns in response to Piscirickettsiosis differentially expressed genes (DEG) among control and infected conditions, were obtained in pairwise comparison with the R package EBSEA v 1.26.0 [33]. In contrast with the conventional gene-level counts approach, EBSEA utilize an exon-level counts with an empirical Brown's based aggregation method, to calculate DEG. Regarding AS, the exon-level counts method performs better than conventional gene-level counts [34]. Genes with a FDR p-value <0.01 were considered as DEG.

### 1.4 Functional gene enrichment

Functional enrichment analysis of DSG was performed on KEGG (Kyoto Encyclopedia of Genes and Genomes) pathways and biological process and molecular function GO (Gene Ontology) terms, with the R package Cluster Profile v 4.2.2 [35] and with the Zebrafish (*Danio rerio*) reference genome. The Zebrafish, is the most functionally annotated fish model. KEGG pathways and GO terms were treated as significant with a Fold Enrichment >2 and an FDR p-value <0.05. In addition, due to the lack of a specific salmonids database of splicing factors (SF), heterogeneous nuclear ribonucleoproteins (hnRNP), and serine-arginine proteins (SR), we used the dataset developed for Zebrafish by Liu et al. [36].

### 1.5 RT-qPCR validation of AS events

To validate differentially expressed inclusion DASE-isoforms, three DSG (serine/arginine-rich splicing factor 7, splicing factor U2AF 35 kDa subunit and programmed cell death 6-interacting protein) were choose for RT-qPCR analysis. Head kidney tissue samples were obtained from six healthy and naturally infected *P. salmonis* Atlantic salmon organisms, by standard field health scrutiny. Total RNA was isolated employing the TRIzol reagent (Invitrogen) and purified using the miRNeasy Mini Kit (Qiagen) and an on-column DNase I treatment. Purity, RNA concentration and integrity were determined by spectrophotometry (BioPhotometer, Eppendorf) and Agilent Bioanalyser 2100, respectively. Specific primers for each AS category, were designed by PrimerSeq v 1.1 [37]. cDNA was synthesized using SuperScript III (Thermo Fisher) and oligo (dT) primers (Thermo Fisher) and qPCR were carried out using SYBR Green (Thermofisher Scientific) and Rotor-Gene Q (Qiagen) platform. All the qPCR reactions were performed in triplicates and two genes, eifed and rpl32, were utilized as normalizers [38]. Thermal cycling was performed as follows: 94 °C for 4 min, followed by 35 cycles of 94 °C for 15 s, 52–58 °C for 30 s and 72 °C for 35 s and a last extension step of 72 °C for 10 min. Each primer pair's RT-qPCR primer efficiency was assessed using 10-fold serial dilutions of cDNA selected from the samples. The Pfaffl method [39] and the Mann-Whitney *U* test in R (R Development Core Team), were used to determine the relative expression of the DSG selected. Differences with a p-value <0.05 were considered as statically significant.

## 22.1 Results Overview of sequencing statistics

In the current study, a total of 1,571,503,661 raw paired-end reads were acquired, covering 59 head kidney samples, with an 97 %, on average, of which were observed as clean reads after quality checking. All clean reads were aligned to the Atlantic salmon reference genome (Ssal v3.1) for ASE, DASE and DEG. On average, 21,393,527 reads were uniquely mapped to a unique Atlantic salmon genomic region (Table S1).

### 2.2 Global characterization of head kidney ASE in response to piscirickettsiosis

**Fig. 1:**
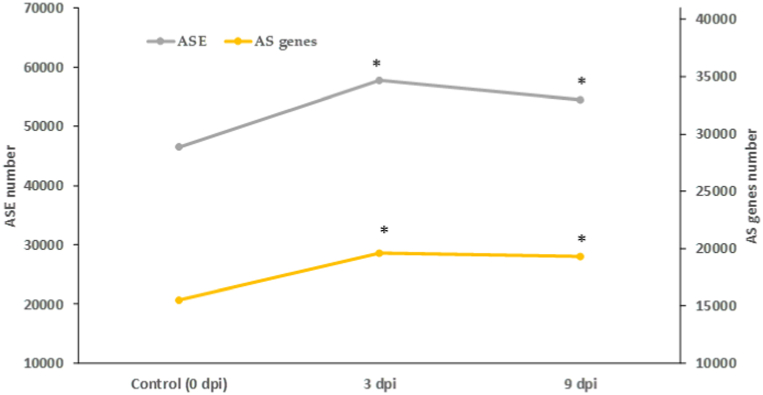
Dynamics changes of the number of ASE and alternatively protein-coding spliced genes after Atlantic salmon *P. salmonis* infection. dpi: days post-infection; *: significant increase with regard to control (0 dpi), chi-square test, p < 0.05.

To examine the global ASE response of Atlantic salmon infected with *P. salmonis* the head kidney RNA datasets (59 samples) were grouped into three independent conditions, control (pre-challenged, 0 dpi) and infected 3 and 9 dpi (post-challenged). A total of 158,668 ASEs, corresponding to 21,510 alternatively protein-coding spliced genes were detected, considering both infected and control groups. The specific dynamics of change of ASE and alternatively protein-coding spliced genes, during the progression of the disease, showed that after the *P. salmonis* challenge, the number of ASE and alternatively protein-coding spliced genes both expanded rapidly at 3 dpi. As the disease continue (9 dpi), the levels of ASE and alternatively protein-coding spliced genes decreased slightly, but held on higher levels than the control (0 dpi) (Fig. 1; Table S2).

**Fig. 2:**
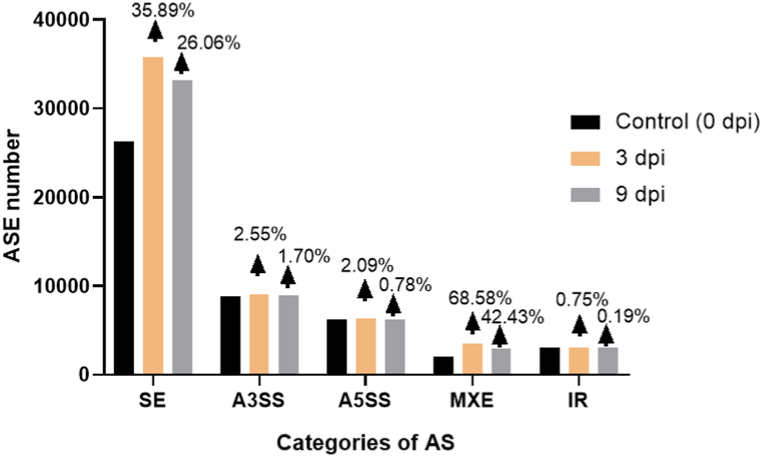
AS identified categories of control and Atlantic salmon conditions challenged with *P. salmonis*. (A) The aim AS categories analyzed: mutually exclusive exons (MXE), intron retention (IR), skipped exon (SE), alternative 3′ splice sites (A3SS) and alternative 5′ splice sites (A5SS). (B) Number of total ASE established on AS categories. Black arrows indicate percentage of increment with regard to control.

An analysis by AS categories, showed that the five AS categories were identified in control and infected conditions with similar patterns. Likewise, the observed frequencies of ASE by AS categories, were homogeneous among the different infected conditions (2.31–1.41 ASE/gene on average) (Fig. 2A and B; Table S2). A specific analysis of ASE among the three conditions (control, 3 dpi and 9 dpi), identified the SE category as the most frequent (mean: 59.85 % ± 2.83) and IR (mean: 5.84 % ± 0.64) and MXE (mean: 5.30 % ± 0.80) as the least abundant, which is coherent with preceding reports [40]. Moreover, after *P. salmonis* infection, all categories of ASE increased, in which for instance, *P. salmonis* induced a 35.89%–26.06 % increase in SE and 68.85%–42.43 % in MXE. These results highlight the substantial and complex AS response of Atlantic salmon to Piscirickettsiosis.

### 2.3 Atlantic salmon differential splicing in response to piscirickettsiosis

A total of 238 and 436 DASEs were recognized in control vs 3 dpi and 9 dpi conditions, which were generated from 191 to 324 DSGs, respectively (Table 1; Table S3). This result show, that AS responses undergoing the 9 dpi condition was intensified compared with the 3 dpi condition. The exon splicing design of the DSG detected was similar to both post infection conditions, with an overriding pattern of exclusion levels in infected conditions. Among the 191 DSGs-3 dpi detected, ES was the most frequent splicing category (58.40 % and 116 genes), followed by MXE (12.18 % and 29 genes), IR (12.18 % and 29 genes), A3SS (10.50 % and 25 genes) and A5SS (6.72 % and 16 genes). In the case of the 324 DSGs-9 dpi observed, also ES (64.81 % and 225 genes) and MEX (12.61 % and 41 genes) were the most frequent splicing categories, but followed by A5SS (7.80 % and 32 genes), IR (7.34 % and 31 genes) and A3SS (7.34 % and 31 genes).

**Table 1:** DASE in control and Atlantic salmon conditions challenged with *P. salmonis*. The numbers differentiated by a semicolon indicate the DASE number with higher inclusion measurements in the control vs days post infection (dpi) conditions.

AS events	Control vs 3 dpi	Control vs 9 dpi
Alternative 5′ splice site	16 (7;9)	34 (19;15)
Alternative 5′ splice site	25 (11;14)	32 (14;18)
Mutually exclusive exon	29 (14;15)	55 (34;21)
Intron retention	29 (6;23)	32 (14;18)
Exon skipping	139 (68;71)	283 (135;148)
Total	238 (106;132)	436 (216;220)

An analysis of the DSG observed at both infected conditions, showed that several of them involved coding proteins with relevant roles in mRNA splicing, such as five hnRNPs (e.g., hnRNP m, hnRNP d and hnRNP c), ten SPs/SRs (e.g., U2AF1, SPF45 and SRSF7), seven RNA binding proteins (e.g., RNA-binding protein 4.1-like, RNA-binding protein 5-B and LUC7-like) and eleven RNA splicing associated proteins (e.g., WD repeat domain 55, YTH domain-containing family protein 1 and PRP39 pre-mRNA processing factor 39 homolog) (Table 2; Table S4).

**Table 2:** RNA splicing associated DSGs among control and *P. salmonis* infected conditions of Atlantic salmon.

Gene ID	Gene Description
106568347	heterogeneous nuclear ribonucleoprotein K-like
100194558	heterogeneous nuclear ribonucleoprotein D-like
106568327	heterogeneous nuclear ribonucleoprotein D
100195139	heterogeneous nuclear ribonucleoprotein M
106569367	heterogeneous nuclear ribonucleoprotein C
106582147	splicing factor U2AF 35 kDa subunit
100195071	splicing factor, arginine/serine-rich 16
106576190	splicing factor 45
100380860	splicing factor 3B subunit 1
106583084	serine/arginine-rich splicing factor 3
106573861	serine/arginine-rich splicing factor 11
106564788	serine/arginine-rich splicing factor 7
106568192	serine/arginine-rich splicing factor 9-like
106578667	poly(U)-binding-splicing factor PUF60-like
106582447	muscleblind-like protein 2a
123723714	RNA-binding protein 4.1-like
100380672	RNA-binding protein 5-B
106579244	LUC7-like (*S. cerevisiae*)
106573877	DAZ associated protein 1
106565996	TAR DNA binding protein a
106565016	RNA-binding protein 39
106570934	zinc finger RNA-binding protein
106571578	PRP39 pre-mRNA processing factor 39 homolog (yeast)
100380337	cold shock domain containing E1, RNA-binding

**Fig. 3:**
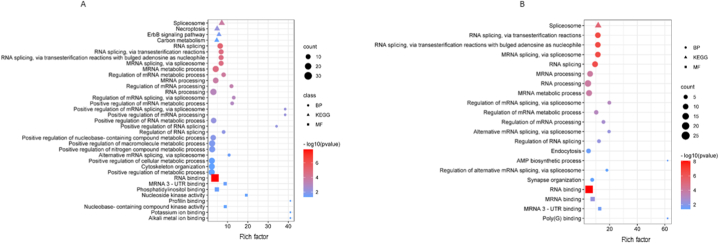
Functional enrichment analysis of DSG of Atlantic salmon in response to *P. salmonis* infection. (A) GO and KEGG enrichment analysis of DSG in 3 dpi condition. (B) GO and KEGG enrichment analysis of DSG in 9 dpi condition.

To gain insight on the biological functions of the obtained DSG, GO and KEGG functional gene enrichment analyses were performed at 3 dpi and 9 dpi conditions, independently. A total of 20 (16 biological processes and 4 molecular functions) and 31 (23 biological processes and 8 molecular functions) GO terms were significantly enriched (Fold enrichment >2 and FDR p-value <0.05) for the 3 dpi and 9 dpi conditions, respectively (Fig. 3A and B). Notably in both conditions, the GO biological processes terms revealed a strong enrichment for different GO terms associated to RNA processing, splicing and spliceosome related genes (e.g., RNA processing: GO:0006396; mRNA processing: GO:0006397; RNA splicing via spliceosome: GO:0000398; RNA splicing via transesterification reactions with bulged adenosine as Nucleophile: GO:0000377 and RNA splicing via transesterification reactions: GO:0000375). These GO enriched terms suggest that *P. salmonis* infection change the splicing pattern of splicing/spliceosome-related Atlantic salmon genes. In addition, several metabolic GO biological process terms also were significantly enriched (e.g., AMP biosynthetic process: GO:0006167; Positive regulation of RNA metabolic process: GO:0051254 and Positive regulation of nucleobase-containing compound metabolic process: GO:0045935). Several studies have highlighted the relevance of metabolic processes in the development of an adequate immunological response [41]. The significantly enriched molecular function GO terms allocated to the DSG were associated with different binding processes, including RNA and MRNA binding. Furthermore, KEGG analysis shown that spliceosome, necroptosis, ErbB signaling and carbon metabolism were enriched pathways in the DSG.

### 2.4 Comparative analysis of atlantic salmon DEG and DSG in response to piscirickettsiosis

**Fig. 4:**
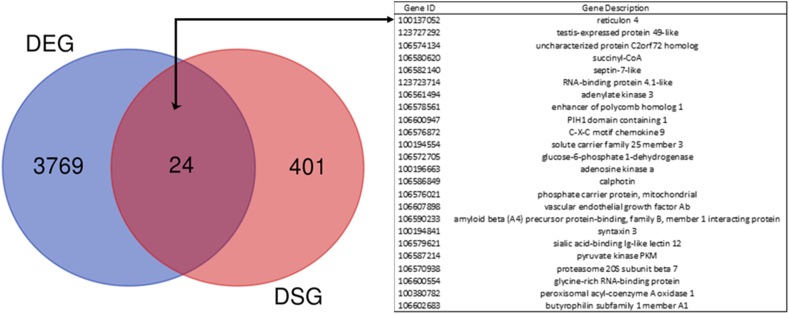
Venn diagram displaying the overlap of differentially spliced (DSG) and differentially expressed genes (DEG) genes, in Atlantic salmon in response to *P. salmonis* infection.

To evaluate the relationship between transcriptional and AS regulation we contrast the genes experiencing transcriptional and AS changes in both infected conditions. EBSEA results showed a total of 3793 DEGs (1447 and 3510 in 3 dpi and 9 dpi conditions, respectively) (Table S5). A comparison among DSG and DEG determined that DSG were normally not differentially expressed, with scarcely 5.65 % of DSG being DEG. The shared DGS-DEGs (24) were associated fundamentally to metabolic and cellular processes (Fig. 4). Furthermore, an analysis of the DEG achieved by Moraleda et al. [27] with the DSG obtained in the present study, also show a reduced overlap among them (7.6 %).

### 2.5 Validation using quantitative real time PCR

**Fig. 5:**
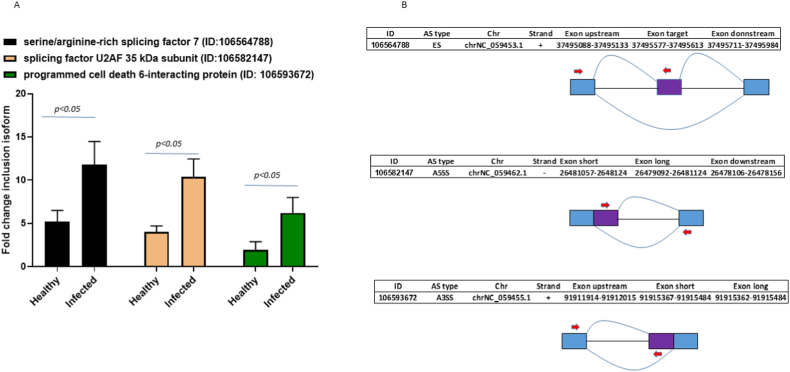
qPCR validation of selected DASE of head kidney tissue from healthy and naturally infected *P. salmonis* Atlantic salmon organisms. (A) Three ASE were selected for RT-qPCR, representing skipped exon (SE), alternative 3′ splice sites (A3SS) and alternative 5′ splice sites (A5SS), events. (B)Transcript structure of corresponding selected DSG. Boxes represent exons, straight lines indicate introns, flexed lines constitute splice junctions and red arrows indicate primers positions.

Three DSG were chosen as DASE model for qPCR validation. The serine/arginine-rich splicing factor 7 (LOC106564788) and splicing factor U2AF 35 kDa subunit (LOC106582147) are widely accepted genes with crucial functional roles in alternative and constitutive splicing process. Programmed cell death 6-interacting protein (LOC106593672) is a recognized gene with versatile functions, implicated in programmed cell death processes to combat infections [42]. The efficiency of all designed primer pairs ranged from 94.31 to 97.56 %, within of the optimal efficiency range recommended for qPCR analysis [43]. Standard curves showed high linearity, with correlation coefficients (R^2^) ranging from 0.95 to 0.98 (Table S6) The relative expression of DASE inclusion isoforms in healthy and *P. salmonis* infected organisms was in agreement with RNA-seq results (Fig. 5A and B).

### 2.6 Phenotype-specific patterns of differential splicing in response to piscirickettsiosis

**Fig. 6:**
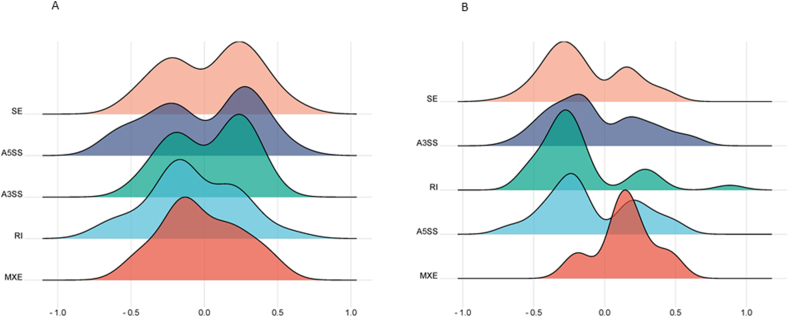
Density plot comparison of the five particular categories of ASE assessed in the present investigation (SE, skipped exon; A3SS, alternative 3′ splice sites; A5SS, alternative 5′ splice sites; MXE, mutually exclusive exons and IR, intron retention) in resistant vs susceptible-3 dpi (A) and resistant vs susceptible-9 dpi (B) Atlantic salmon conditions.

**Fig. 7:**
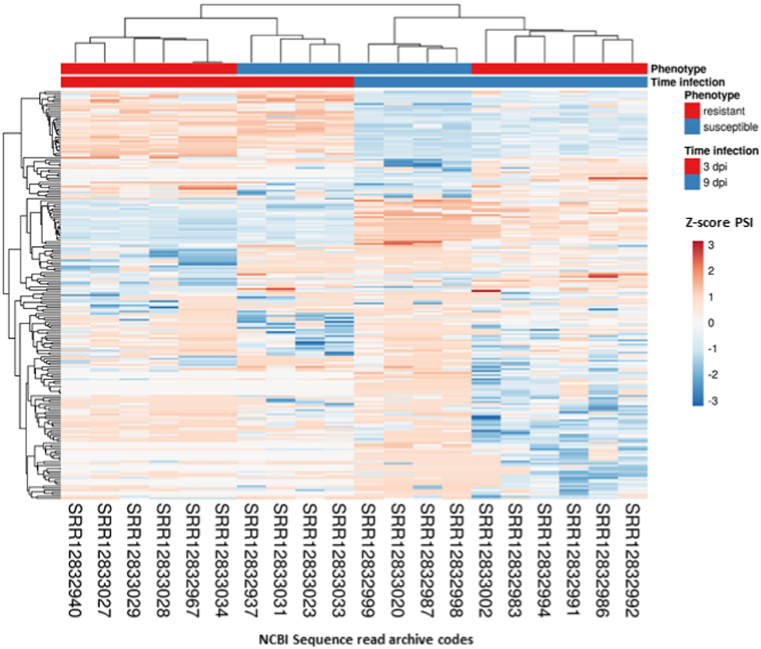
Heatmap clustering of DASE among resistant and susceptible 3 dpi and 9 dpi Atlantic salmon phenotypes. The progressive color variation from orange to blue, represents Z score PSI levels.

To investigate the effect of genetic background of the dynamics of DASE, resistant and susceptible *P. salmonis* Atlantic salmon phenotypes were selected from the RNA-seq set, based on EBV. A total of 168 and 199 DASEs were identified among resistant vs susceptible 3 dpi and resistant vs susceptible 9 dpi phenotypes, which were generated from 153 to 177 DSGs, respectively. The SE category was the most dominant (3 dpi: 36.1 % and 9 dpi: 37.6 %) and IR (3 dpi: 17.8 and 9 dpi: 4.7 %) and MXE (3 dpi: 4.1 % and 9 dpi: 3.0 %) being the least common (Table S7). In addition, a preponderant pattern of inclusion levels was observed in resistant samples in 3 dpi, which was switched to a dominant pattern of exclusion levels in 9 dpi (Fig. 6A and B). Furthermore, a heatmap clustering on the standardized PSI values, revealed noticeable clusters according to phenotypes and times of infection (Fig. 7).

**Fig. 8:**
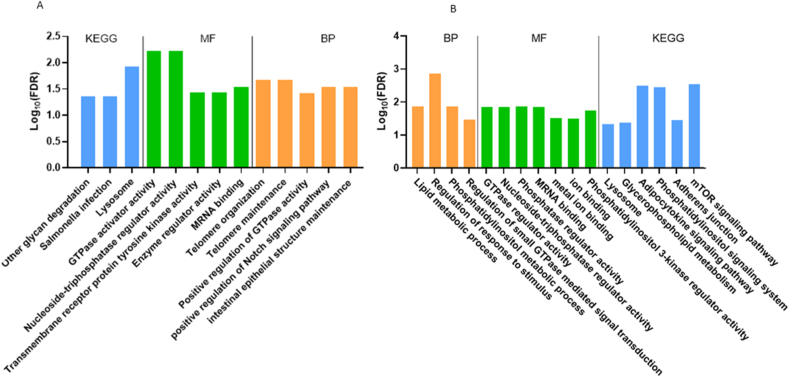
GO and KEGG enrichment analysis of DSG in resistant vs susceptible Atlantic salmon. GO biological process (BP), molecular function (MF) and KEGG enrichment analyses of resistant vs resistant vs susceptible-3 dpi (A) and resistant vs susceptible-9 dpi (B) Atlantic salmon conditions.

To further to evaluate the biological functions of AS among resistant/susceptible phenotypes, we gathered the overlapping DSG between the resistant/susceptible and control/infested comparisons, to create a set of exclusive resistant/susceptible phenotypes-DSG. After excluding overlapping DSG, a total of 150 and 162 DSGs were made unique to the 3 dpi and 9 dpi resistant/susceptible groups, respectively. To investigate the biological functions of the resistant/susceptible DSG groups, GO and KEGG functional gene enrichment analysis were performed at 3 dpi and 9 dpi conditions, independently (Fig. 8A and B).

First, we recognized a total of nine biological process (BP) terms enriched, overlapping both conditions. The BP terms enriched were related mainly with cellular lipid metabolic (e.g., Phosphatidylinositol metabolic process: GO:0046488), regulation of molecular function (e.g., Positive regulation of GTPase activity: GO:0043547) intracellular signal transduction (e.g., regulation of small GTPase mediated signal transduction: GO:0051056), chromosome organization (e.g., telomere organization: GO: GO:0032200), cell surface receptor signaling (e.g., positive regulation of Notch signaling pathway: GO: 0045747), and digestive system (intestinal epithelial structure maintenance: GO: 0060729) processes. Notably, recent studies have suggested a relevant relationship between telomere maintenance and an appropriate immune response in vertebrates [44]. In addition, ten molecular function enriched terms were recognized in both conditions, associated mostly to enzyme regulator (e.g., Transmembrane receptor protein tyrosine kinase activity: GO:0004714; Nucleoside-triphosphatase regulator activity: GO:0060589; GTPase regulator activity: GO:0030695) and ion binding (e.g., metal ion binding: GO: 0046872) activities. In the context of KEGG enriched pathways, several relevant immune pathways were recognized such as Lysosome (ko:04142), Adipocytokine signaling pathway (ko:04920), Adherens junction (ko: 04520) and mTOR signaling pathway (ko: 04150). These findings show that AS regulation in resistant/susceptible *P. salmonis* Atlantic salmon phenotypes is not an arbitrary procedure, rather it is connected to contrasting phenotypes responses.

## 3 Discussion

Throughout their development processes and growth, salmonids are exposed to a variety of pathogen and environmental challenges. To manage these pressures, they have developed complex mechanisms at both the transcriptional and post-transcriptional level. Nonetheless, the present-day comprehension of salmonids transcriptome responses to pathogen infection is largely focused on transcriptional responses, whilst post-transcriptional processes of gene regulation are much less understood. AS has been clearly shown to perform crucial roles in the response of animals and plants to combat bacterial infections, at post-transcriptional level [45] however, is unknown how AS contributes to the salmonids bacterial infection responses. Therefore, we can hypothesize that bacterial infections will significantly affect the Atlantic salmon AS regulation. Consequently, in the present work, we performed a genome-wide study of AS in the head kidney tissue of Atlantic salmon challenged with *P. salmonis*, based on 59 paired short-read RNA sequenced (RNA-seq) samples. Paired sequencing, is a relevant approach in the context of AS analyses as they strictly map reads and recognize splice junctions, allowing a correct estimation of alternative spliced isoforms [46,47]. However, from a theoretical point view the sequencing of a whole gene, from the beginning to end, will make it possible to determine all its isoforms. Notwithstanding, RNA-seq ASE results are built on exact modelling premises, specific processing and analytic methods [48]. Thus, paired RNA-seq has led the identification and quantification of ASE in a high number of plant and animal species.

Foregoing studies have shown the relevance of the AS process in the modulation of patterns of gene expression in mammals infected with intracellular bacteria [21]. Our findings in this study indicated that *P. salmonis* infection significantly increased the overall number of AS events and alternatively protein-coding spliced genes at both 3 dpi and 9 dpi conditions. Interestingly, recent studies in catfish challenged with another gram-negative bacteria *Edwarsiella ictaluri* and *Flavobacterium columnare* [25,26], also have revealed modifications of AS patterns similar to those observed in the present work. Therefore, AS process seems to be crucial to the regulation of gene expression following *P. salmonis* infection in Atlantic salmon. A total of 238 and 436 *P. salmonis*-responsive DASEs were identified in 3 dpi and 9 dpi Atlantic salmon challenged conditions, respectively. Although all the main five AS categories were identified in these DASEs, SE was the most frequent *P. salmonis* responsive AS category in both conditions (average SE: 61.05 ± 4.53). This is consistent with the reported to catfish in the course of *E. ictaluri* and *F. columnare* infections [25,26]. Thus, the SE category seems to be the category dominant in the differential responsive AS categories of teleost, under bacterial infections. GO and KEGG gene enrichment analysis of DSG associated with these *P. salmonis*-responsive DASE in both conditions, 3 dpi and 9 dpi, unveiled that splicing and spliceosome genes were overrepresented and that they involved many types of splicing-associated genes (e.g., splicing factor U2AF 35 kDa subunits, serine/arginine-rich splicing factor 7, heterogeneous nuclear ribonucleoprotein M, RNA-binding protein 39). These genes have been reported as important components in the regulation of post-transcriptional modifications in diverse physiological conditions and diseases [49,50]. The splicing of splicing-associated genes will potentially affect the configuration of the spliceosome, affecting in turn, the splicing of several downstream elements, such as genes activated during bacterial infections. Hence, AS may represent an important molecular process for Atlantic salmon to diversify its transcriptome in response to *P. salmonis* infections.

Previous transcriptomic studies evaluating the responses of *P. salmonis* infection in Atlantic salmon, have reported several upregulated immune genes involved in several immune pathways such as apoptosis, Toll-like receptor signaling, and cytokine-cytokine receptor [51]. Notably, a limited number of immune-associated DSG (e.g., CD209 antigen-like protein and suppressor of cytokine signaling 3-like) were observed in the present study. This led us to evaluate in detail the relationships between Atlantic salmon DEG and DSG, in response to *P. salmonis*. Comparative analysis shows that DSG were not normally differentially expressed. Theoretically, AS and gene expression could function separately, performing redundant or conflicting roles by impacting the same or different genes-biological pathways, respectively [52,53]. In addition, as AS is presumably a less restrained molecular process than expression modification [54], this can to alter the expression of genes with strong functional constraints, without modifying its originals expression patterns. Our results support the notion that Atlantic salmon combat Piscirickettsiosis by involving both AS and the differential expression mechanism in parallel, albeit in a functionally contrasting but cooperative manner, involving distinct genes and biological pathways.

In salmonids, the transcriptomic analysis of contrasting disease phenotypes, such as resistant and susceptible organisms, has been an issue of constant interest. Here, a total of 150 and 162 DSGs were determined as exclusive to the 3 dpi and 9 dpi resistant/susceptible groups, respectively. GO and KEGG enrichment analysis revealed several functional terms and pathways categories, out of which GTPase regulation, lysosome and telomere organization-maintenance stand out. In the present study, various resistant-susceptible differentially spliced associated GTPases genes (e.g., rho GTPase-activating protein 27 and 12), were identified, which are crucial players in the regulation and formation of actin cytoskeleton [55]. In addition, several resistant-susceptible DSG associated to the lysosome pathway (e.g., lysosome C, AP-1 complex subunit sigma-3-like) were also identified. Notably, both GTPases and lysosomes, are recognized targets of intracellular bacteria through of the secretion of effectors, to maximize its growth and spreading in the host [56,57]. Therefore, AS of GTPases and lysosomes associated genes will be increase after *P. salmonis* infection, in resistant Atlantic salmon, to diversify their transcripts/isoforms, thus reinforcing the adequate molding of actin cytoskeleton and lysosome performance.

Telomere organization-maintenance (e.g., attrition) may be significantly affected by exposure to oxidative stress, inflammation and pathogens [58]. Thus, individuals with long telomeres would show an improved capacity for cell proliferation developing, in turn, a better response to combat bacterial infections. Further studies are required to address whether *P. salmonis* resistant Atlantic salmon fish, present longer telomeres that susceptible organism in for example, macrophages cells.

## Funding

This research was supported by CORFO-INNOVA Chile 20CVC-127999.

## Data availability statement

The RNA-seq data No. PRJNA669807 were downloaded from Sequence Read Archive (NCBI). All data acquired and analyzed in this study are included in this paper and the supplementary information file.

## Ethical approval

Not applicable.

## Consent for publication

All authors agree with submitting the paper for publication in the journal, Heliyon.

## CRediT authorship contribution statement

**Scarleth Bravo:** Conceptualization, Formal analysis, Investigation, Methodology, Software, Writing – original draft, Writing – review & editing. **Javier Moya:** Conceptualization, Formal analysis, Funding acquisition, Investigation, Validation, Writing – original draft, Writing – review & editing. **Francisco Leiva:** Conceptualization, Data curation, Formal analysis, Investigation, Writing – original draft, Writing – review & editing. **Osiel Guzman:** Conceptualization, Formal analysis, Funding acquisition, Methodology, Writing – original draft, Writing – review & editing. **Rodrigo Vidal:** Conceptualization, Data curation, Formal analysis, Funding acquisition, Investigation, Methodology, Writing – original draft, Writing – review & editing.

## Declaration of competing interest

The authors declare the following financial interests/personal relationships which may be considered as potential competing interests: RODRIGO VIDAL reports a relationship with 10.13039/100009465Production Development Corporation (CORFO) that includes: funding grants.
